# Supplementary material for: Identification of photoperiod-induced specific miRNAs in the adrenal glands of Sunite sheep (Ovis aries)
Source: Front Vet Sci. 2022 Jul 22;9:888207. doi: 10.3389/fvets.2022.888207 (PMC9354845; doi:10.3389/fvets.2022.888207)
Supplement: Supplementary file 1 [file Data_Sheet_1.ZIP › Supplementary file/miRDeep2 file.zip!/miRDeep2 file/Novel_Structure/SPLP42A1/Novel_112.pdf]

Provisional ID	:	NC_019482.2_30604
Score total	:	1.7
Score for star read(s)	:	-1.3
Score for read counts	:	0
Score for mfe	:	1.4
Score for randfold	:	1.6
Score for cons. seed	:	
Total read count	:	106
Mature read count	:	106
Loop read count	:	0
Star read count	:	0

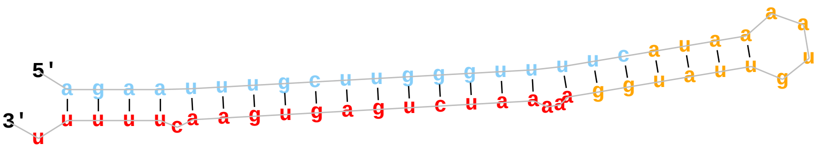

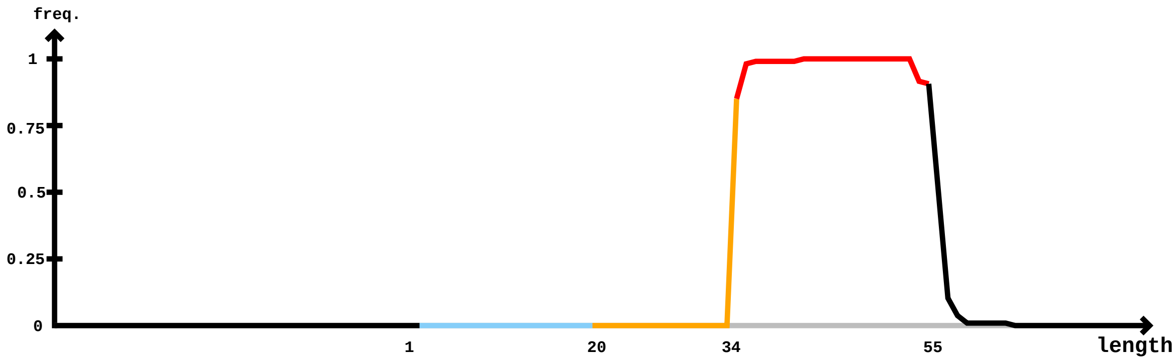

Star Mature

```
5'- aaccauuauauacagaugauuuagugaguuggccaaagaauuugcuuggguuuucauaaaaauguuauggaaaaaucugagugaacuuuuuggccaacggaauauuuuucucc   -3'    exp
    ...(((((.............))))).((((((((((((.((((((((((((((((((((....)))))))..))))))))))))).))))))))))))((((.....))))..    reads      mm          sample
    ..........................................................................aaaaaucugaAugaacuuu........................    9          1           seq
    ..........................................................................aaaaaucugagugaacuuuu.......................    1          0           seq
    ..........................................................................aaaaaCcugagugaacuuuuu......................    1          1           seq
    ..........................................................................aaaaaucugaAugaacuuuuu......................    80         1           seq
    ...........................................................................aaaaucugagugaacuuuuu......................    5          0           seq
    ...........................................................................aaaaucugagugaacuuuuugg....................    6          0           seq
    ...........................................................................aaaaucugaAugaacuuuuuggc...................    2          1           seq
    ...........................................................................aaaaucugagugaacuuuuuggc...................    1          0           seq
    ............................................................................aaaucugagugaacuuuuugg....................    1          0           seq
    .................................................................................ugagugaacuuuuuggccaacU..............    1          1           seq
```
